# Bronchial stenosis requires vigilance against tracheal mucormycosis: a case report

**DOI:** 10.3389/fmed.2025.1510292

**Published:** 2025-03-24

**Authors:** Ye Wang, Shiyao Wang, Hongmei Zhao, Chen Wang

**Affiliations:** ^1^Beijing University of Chinese Medicine, Chaoyang, Beijing, China; ^2^National Respiratory Medical Center, China-Japan Friendship Hospital, Beijing, China; ^3^Peking Union Medical College, Chinese Academy of Medical Sciences, Beijing, China

**Keywords:** mucormycosis, bronchial stenosis, diabetes, posaconazole, pulmonary

## Abstract

This report highlights the importance of differentiating the etiology of dyspnea and tracheal stenosis. While acknowledging that tracheal stenosis can significantly cause respiratory distress, we emphasize the need to consider tracheal mucormycosis in the differential diagnosis, particularly in patients with risk factors such as uncontrolled diabetes and trauma. Early recognition and timely antifungal therapy remain crucial in managing this potentially fatal infection. Prioritizing early identification and treatment of mucormycosis before it progresses to the lungs can significantly improve patient outcomes and impact clinical practice.

## Introduction

Bronchial stenosis, or airway narrowing, is a common clinical presentation with diverse etiologies, ranging from benign inflammatory conditions to malignant tumors. Although most cases are effectively managed with conventional treatments, a subset of patients presents with atypical features or fails to respond to standard therapies, underscoring the importance of considering less common but potentially life-threatening diagnoses. Although mucormycosis, a rare but aggressive fungal infection, is often associated with rhinocerebral manifestations, pulmonary involvement can also occur, potentially leading to bronchial stenosis.

## Case presentation

**Figure 1:**
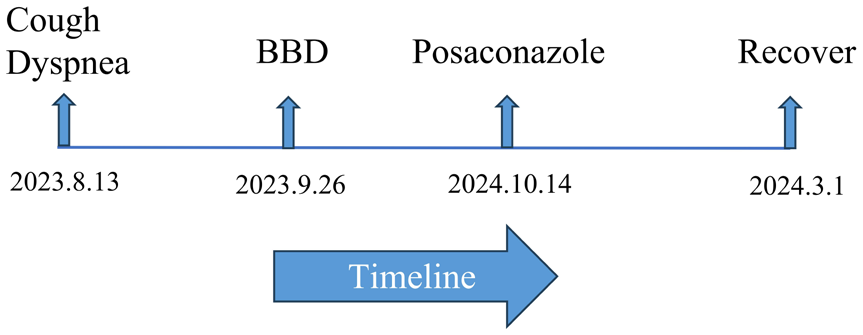
A figure showcasing a timeline with patient.

A 59-year-old male patient presented to our hospital with intermittent cough and progressive exertional dyspnea lasting for 2 months. The patient’s dyspnea primarily manifested as shortness of breath on exertion, with a subjective sensation of breathlessness at rest, which was partially relieved by supplemental oxygen. He had been diagnosed with type 2 diabetes mellitus 10 years earlier and had poor glycemic control. Despite treatment with subcutaneous aspart and glargine insulin, glycemic control remained suboptimal, with fasting glucose levels around 11.0 mmol/L and postprandial glucose ranging from 15 to 18 mmol/L. Due to long-term diabetes mellitus, the patient exhibited compromised renal function upon admission. He also had a history of hypertension for over 10 years, which was well-controlled with oral antihypertensive medication, and a history of herpes zoster for over 1 year. The patient initially presented to a local hospital where a chest CT scan revealed left main bronchial stenosis. Fiberoptic bronchoscope examination with biopsy and BALF analysis for infectious agents and pathology were performed. The results were unremarkable. The patient underwent one session of bronchial balloon dilation at the local hospital before being discharged with the recommendation for a second dilation at a later date. He was then admitted to our hospital for further evaluation and treatment (see Figure 1).

**Figure 2:**
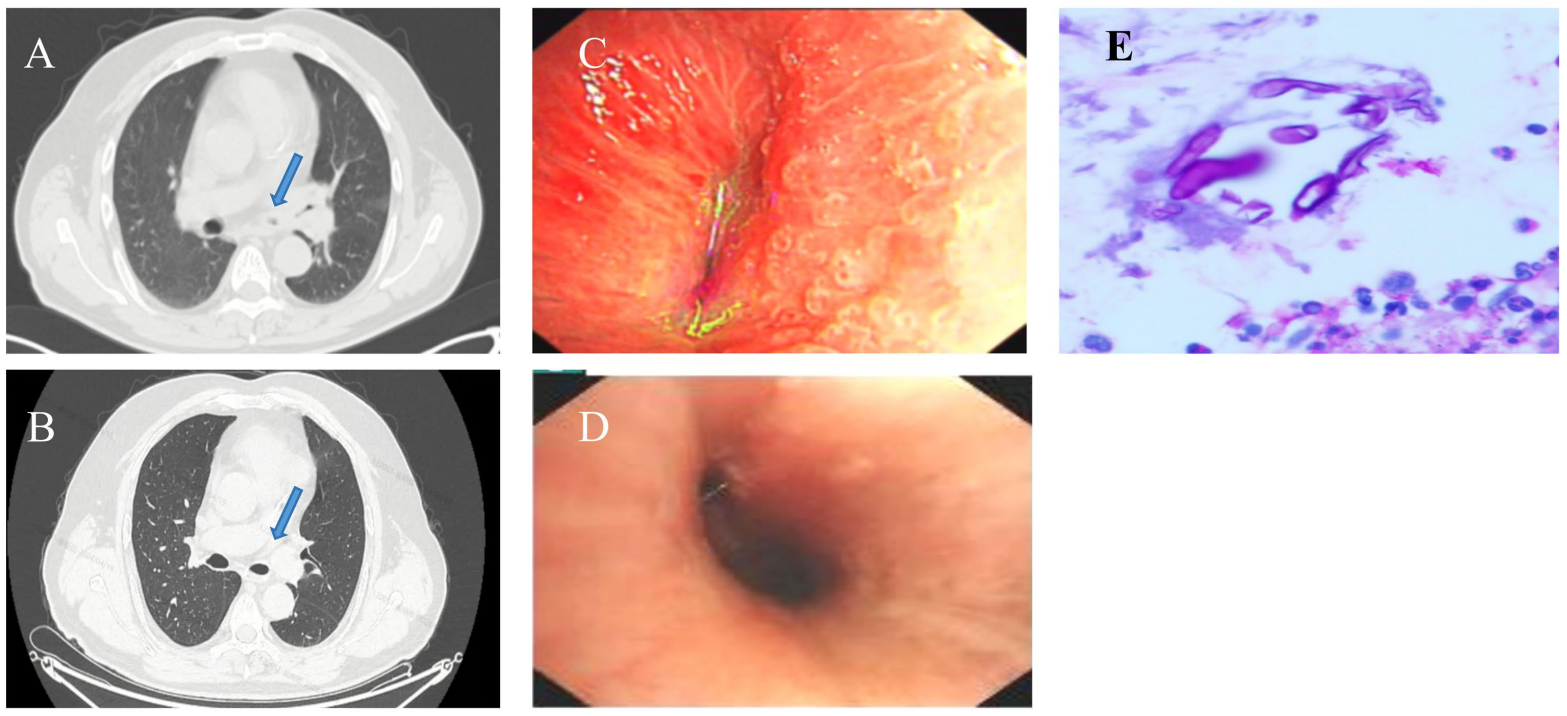
**(A)** PET-CT scan showing thickening and metabolic activity increase in the wall of the left main. **(B)** Follow-up chest CT scan showing significant improvement in bronchial stenosis. **(C)** Bronchoscopy image showing localized swelling and narrowing of the left main bronchus. **(D)** Follow-up bronchoscopy image showing significant improvement in the lumen. **(E)** Pathological examination of EBUS-TBNA specimen showing suppurative granulomatous inflammation with degenerate fungal hyphae consistent with mucormycosis (and positive PAS staining in our hospital).

On physical examination, breath sounds were clear bilaterally, and peripheral oxygen saturation was low. Laboratory investigations revealed a glycosylated hemoglobin (HbAlc) level of 10.5%. Arterial blood gas analysis showed a pH value of 7.417, PaO_2_ of 65.3 mmHg, and PaCO_2_ of 37.9 mmHg (FiO_2_ = 0.21). Inflammatory markers were within the normal range. The urine microalbumin/urine creatinine ratio was 40.6 mg/g, and urine creatinine was 18945.6 μmol/L. Serum creatinine was 78.3 μmol/L. The estimated glomerular filtration rate (eGFR) was 93.9 mL/min/1.73 m^2^. PET-CT revealed thickening and increased metabolic activity in the wall of the left main and left upper lobe bronchus, suggesting the need for fiberoptic bronchoscopy (Figure 2A). The bronchoscopy revealed localized swelling in the distal left main bronchus and left upper lobe bronchus, with thickened and swollen mucosa, severely narrowing the lumen (Figure 2C). Bronchoalveolar lavage was performed in the left upper lobe and sent for infectious agent testing and cell differential counts. The sputum, BALF, brushings, and biopsy specimens were sent for infectious agent analysis. All tests were negative. Endobronchial ultrasound-guided transbronchial needle aspiration (EBUS-TBNA) was performed on the left main bronchial wall, with four specimens sent for infectious agent testing and pathology. The pathology report revealed suppurative granulomatous inflammation with degenerate fungal hyphae, suggesting mucormycosis (Figure 2E). Both serum G and GM tests were negative. The patient was at high risk for fungal infections due to his history of poorly controlled type 2 diabetes mellitus. Based on the pathological findings, a diagnosis of tracheal mucormycosis was established. Amphotericin B was the initial treatment of choice, but due to the patient’s renal impairment and the fact that the mucormycosis was confined to the bronchus, the potential side effects of the drug were deemed too high. Therefore, posaconazole was chosen for antifungal treatment. The treatment was initiated with intravenous posaconazole 300 mg daily for 3 days (with a 600 mg loading dose on day 1), followed by a transition to oral posaconazole enteric-coated tablets 300 mg once daily for ongoing antifungal maintenance. Currently, subcutaneous injections of aspart insulin and glargine insulin are used to control the patient’s blood sugar. The inadequate glycemic control at home was due to the patient’s failure to adhere to the prescribed treatment regimen. Two months later, a follow-up chest CT scan revealed significant improvement. The patient was scheduled for a follow-up evaluation 12 weeks after initiating the posaconazole therapy, but due to travel constraints, they were not seen until 5 months later. At that time, the patient had fully recovered (Figures 2B,D). The patient was contacted monthly by telephone and reported feeling well during each call. Informed consent was obtained from the patient.

## Discussion

All-cause mortality rates for mucormycosis range from 40 to 80%, with varying rates depending on underlying conditions and sites of infection (1). According to the literature (%^2^–^5^), pulmonary mucormycosis (PM) can manifest as nodules, consolidation with halo signs, and even rib fractures (6). However, the patient’s lungs were not affected, and the presentation was instead characterized by bronchial stenosis accompanied by irreversible dyspnea. Extensive BALF and tissue biopsy at the referring hospital yielded no diagnostic clues. Bronchoscopic balloon dilation (BBD) provided only temporary symptomatic relief before the symptoms recurred.

Cases of mucormycosis with only tracheal involvement are relatively rare. Therefore, increasing awareness of tracheal mucormycosis as a differential diagnosis is crucial. Previous studies have reported (7) that bronchoscopy of this disease can reveal granulation tissue (8) and/or gray-white mucinous tissue blocking the main airways, which are often accompanied by edema and necrosis. While biopsy is a crucial diagnostic tool, the likelihood of identifying mucor fungi varies depending on the nature of the tracheal lesion. For patients with localized tracheal necrosis, biopsy is more likely to yield positive results. However, in cases of localized tracheal tissue hypertrophy, multiple biopsies from different sites are often required to obtain a positive result, significantly increasing the diagnostic difficulty (9). This case exemplifies this challenge, as the initial biopsy, which included five tissue samples taken before hospitalization, failed to detect any fungal presence. Despite obtaining four tissue samples from the area of stenosis upon admission, the diagnosis was delayed. This delay was primarily attributed to the atypical nature of the lesion and the low positivity rates in both pathogen and pathological testing.

Previous studies have shown that delayed treatment of mucormycosis increases patient mortality and encourages empirical antifungal therapy prior to histopathology and pathogen culture (10). The main purpose of this report was to differentiate the diagnosis of dyspnea and bronchial stenosis. For patients presenting with only tracheal involvement, especially those with risk factors such as uncontrolled diabetes and trauma, the possibility of tracheal mucormycosis should be carefully considered. This proactive approach facilitates timely diagnosis and allows for the prompt initiation of antifungal therapy, potentially improving patient outcomes.

## Data Availability

The raw data supporting the conclusions of this article will be made available by the authors, without undue reservation.
